# Effects of Social Support by a Dog on Stress Modulation in Male Children with Insecure Attachment

**DOI:** 10.3389/fpsyg.2012.00352

**Published:** 2012-09-28

**Authors:** Andrea Beetz, Henri Julius, Dennis Turner, Kurt Kotrschal

**Affiliations:** ^1^Department of Special Education, University of RostockRostock, Germany; ^2^IEMT SwitzerlandZurich, Switzerland; ^3^Department of Animal Behavior, University of Zurich-IrchelZurich, Switzerland; ^4^Department of Behavioral Biology, University of ViennaVienna, Austria; ^5^Konrad Lorenz Research Station GruenauGruenau im Almtal, Austria

**Keywords:** attachment, human-dog interaction, social support, stress regulation, children, cortisol

## Abstract

Up to 90% of children with special education needs and about 40% of children in the general population show insecure or disorganized attachment patterns, which are linked to a diminished ability to use social support by others for the regulation of stress. The aim of the study was to investigate if children with insecure-avoidant/disorganized attachment can profit more from social support by a dog compared to a friendly human during a stressful task. We investigated 47 male children (age 7–11) with insecure-avoidant or disorganized attachment. Social stress was elicited via the Trier Social Stress Test for Children (TSST-C). For one group of children a friendly therapy-dog (*n *= 24) was present, for one control group a friendly human (*n *= 10) and for the other control group a toy dog (*n *= 13). Stress levels of the children were measured via salivary cortisol at five times (*t*1–*t*5) before, during, and after the TSST-C and subjective reports. The physiological stress response was significantly lower in the dog condition in comparison to the two other support conditions at *t*4, *t*5 and the overall stress reaction from *t*1 to *t*5 (Area Under the Curve increase; Kruskal–Wallis *H*-Test, pairwise *post hoc* comparisons via Mann–Whitney *U*-Tests). Cortisol levels correlated negatively (*r*_s_) with the amount of physical contact between the child and dog. We conclude that male children with insecure-avoidant or disorganized attachment profit more from the presence of a therapy-dog than of a friendly human under social stress. Our findings support the assumption that the increasing practice of animal-assisted education is reasonable and that dogs can be helpful assistants in education/special education, since stress interferes with learning and performance in students.

## Introduction

Largely independent of the quality of the parent-child-relationship, children readily develop trustful relationships with companion animals, and often communicate personal matters to pets rather than to other humans (Kurdek, 2008, 2009a,b; Parish-Plass, 2008). As social companions, animals may benefit the development of children in the cognitive, emotional, and physical domains (Melson et al., 1991; Bodmer, 1998; Melson and Fine, 2006).

Attachment theory provides a particularly powerful background for the explanation of the positive socio-emotional effects of animals, although Ainsworth (1963, 1972) and Bowlby (1969/1982), the founders of attachment theory, did not integrate animals in their deliberations. In particular, attachment theory contributes toward our understanding why many humans in need of social support relate more easily and spontaneously to animals than to other humans (Brown and Katcher, 2001; Beck and Madresh, 2008; Kurdek, 2008, 2009a,b; Beetz et al., 2011). Children develop their specific attachment representation (see below) during their first year of life, mainly via interacting with their primary caregivers (Bowlby, 1969/1982). The primary function of the behavioral system (Baerends, 1976; George and Solomon, 2008; Marvin and Britner, 2008) of attachment is to establish and maintain proximity to the caregiver, ensuring caregiving and protection for the child. Thereby, this behavioral system also reduces and buffers stress, because an effective caregiver also provides a safe haven in stressful contexts and serves as a secure base for exploration of the environment (Bowlby, 1969/1982). Sensitive and reliable early caregiving generally results in secure attachment (Ainsworth et al., 1971). Secondary adaptive strategies such as insecure (avoidant or ambivalent) or disorganized attachment develop in reaction to sub-optimal caregiving. These strategies are regulated via mental representations, so-called “internal working models” (Bretherton and Munholland, 2008), which evaluate and organize the experiences made and affect how individuals respond to their primary caregivers.

In contrast to securely attached children, children with insecure-avoidant attachment have experienced their caregivers as rejecting and unsupportive and therefore, may avoid proximity rather than relating to them when stressed. As an alternative strategy, avoidant children try to distract themselves and tend to emphasize explorative behavior in stressful situations (Ainsworth and Wittig, 1969; Ainsworth et al., 1971), instead of crying or openly seeking proximity and contact. However, their cortisol levels during a separation from their caregivers are generally higher than in securely attached children (Spangler and Schieche, 1998). Attachment disorganization is characterized by a breakdown of adaptive strategies in relevant situations (Main and Solomon, 1986, 1990), which may be reflected in different ways, e.g., dissociation, disorientation, fear, or aggressive behavior, particularly in the context of social stress. Disorganized attachment may develop in response to abusive, negligent, or frightening behavior of the caregiver or in response to loss of, and separation from that person. Insecure as well as disorganized attachment, found in 60–90% of clinical samples or populations in schools for special education (van Ijzendoorn and Bakermans-Kranenburg, 1996; Julius, 2001), are considered risk factors for socio-emotional development (Strauss et al., 2002), while secure attachment is known as a potent protective factor (Werner and Smith, 1982). Also in non-clinical samples only 50% (Grossmann et al., 1981, German sample) to 60% (Ainsworth et al., 1978, US-sample) of the children show a secure attachment.

The internal working model developed with the primary caregiver is normally transferred to all further close relationships (Sroufe and Fleeson, 1988; Howes and Hamilton, 1992; Dozier et al., 2001; Sroufe et al., 2005), for example to teachers or therapists. Hence children with insecure or disorganized attachment find it more difficult than individuals with secure attachment to seek effective social support from other humans and profit from them in stressful situations (Maunder and Hunter, 2001). In general, attachment status and social support are considered major and closely related factors in modulating both the sympatho-adrenergic stress axis as well as the hypothalamo-pituitary-adrenal (HPA) axis, and in adjusting their reactivity early on (de Vries et al., 2003; Beetz et al., 2011; Julius et al., 2013). This may have far-reaching consequences for life, because sub-optimal social relations are currently recognized as a major risk factor for individual well-being and health (Coan, 2011).

This social regulation of stress and the influence of attachment representations can also be linked to other models of stress regulation such as Folkman’s and Lazarus’ transactional model of stress (Lazarus and Folkman, 1984). It can be assumed that a child (or adult) with insecure or disorganized attachment has learned that effective social support as a coping resource is not available in times of distress. Even though other resources (e.g., problem solving skills, utilitarian resources, etc.) might be available to the individual, this learned discouragement of seeking social support and thus diminished availability of social support as stress management strategy might increase the stress via an increased discrepancy between the person’s coping resources and the demands of a given situation.

It is further plausible that insecure and/or disorganized attachment could be linked to deficits in personal self-regulation, as postulated by the DEDEPRO Model (De la Fuente and Justicia, 2007). Good behavioral hetero-regulation (e.g., by the parents) in early childhood, as in a secure attachment promoted by sensitive and available caregivers, would promote the internalization of adaptive self-regulating behavior with adequate coping strategies that focus on social management. A social environment with little or inadequate social hetero-regulation (insecure attachment) in contrast, would favor the establishment of social avoidance strategies or strategies that focus on emotion management (in the sense of emotional down regulation which is typical of insecure-avoidant attachment), but are likely less effective in the regulation of physiological stress responses.

In a school context, an insecure attachment pattern impairs a student’s ability to profit from social support by his teacher or peers in stressful situations with regard to stress regulation. This again, however, negatively influences performance (e.g., in test situations) and cognitive and socio-emotional learning in general, since the prerequisites, such as executive functions (EF, e.g., cognitive flexibility, impulse control, working memory, self-motivation, problem solving, reasoning, and planning; Miyake et al., 2000) located in the prefrontal cortex are sensitive toward stress. Already slight increases in the stress-hormone cortisol are associated with a noticeable decrease in executive functioning.

According to the criteria of Ainsworth(1991; Kurdek, 2008), companion animals can be attachment figures for their owners. Animal owners, and particularly children, indeed frequently turn to their animals for social support in emotionally stressful situations (Rost and Hartmann, 1994; McNicholas and Collis, 2006). Even children with sub-optimal attachment are obviously able to meet companion animals with trust (Kurdek, 2008, 2009a,b), which is consistent with the clinical experience that children are open to relate to pets even if they would not approach human caregivers in stressful situations. Hence, attachment representations acquired with humans are seemingly not spontaneously transferred to animals. In fact, interacting with a friendly companion animal may be associated with lower cardiovascular responses and cortisol levels than interacting with people (Friedmann et al., 1983; Lynch, 1985; Allen et al., 1991, 2002; Odendaal and Meintjes, 2003). Such effects are commonly related to effective social support. Touch and physical contact, as a component of social support (Ditzen et al., 2008), is mainly observed in secure attachment relationships with humans (Hazan and Zeifman, 1999). However, it is a concomitant of interactions with friendly companion animals, especially dogs, and has been shown to significantly contribute to stress attenuation in children with insecure and disorganized attachment (Beetz et al., 2011).

Based on the hypothesis that particularly in children with insecure or disorganized attachment an animal may be a more efficient emotional social supporter than either a friendly adult or a toy dog, we predict that children with such attachment will experience a greater stress-alleviating effect from the presence of a friendly dog than of a friendly person when exposed to a social stressor. Furthermore, we expect that the quality and quantity of interactions between child and social supporter will affect stress modulation. To test these predictions, we exposed children with insecure and/or disorganized attachment to a standardized social stressor, either in the presence of a real dog, a friendly human, or a toy dog as an additional control for the potential specific effects of the presence of a real dog. To judge the effects on the HPA-axis, cortisol was determined from saliva samples. Furthermore, the behavior of children was coded from video tape.

## Materials and Methods

### Sample

Male second to fourth graders (age 7–11 years) were recruited via a regular school and via several schools for children with learning and emotional and behavior disorders in Germany and Austria. The children participated on a voluntary basis. Informed consent was obtained from their legal guardians, the school headmasters and the ministry of education and these documents are archived by the authors. The study was approved by the Human Subjects Review Committee of the University of Rostock, Germany and conforms to the Declaration of Helsinki for experiments on human subjects.

To reduce variability in our sample, only male children were recruited for participation at this time. The majority of previous controlled stress studies including children found no apparent sex-differences in the stress reactions in response to stress-inducing procedures (for a review see Kudielka and Kirschbaum, 2005). Also both, male and female children are able to develop trustful relationships to companion animals. Therefore, it is likely that our findings could be confirmed for female children in the future. Furthermore, the majority of students placed in special education classes due to learning, behavioral, or emotional disorders is male, and for these children the hypothesized positive effects of support by a dog are particularly relevant.

From an original sample of 88 male children, 47 children were selected which could be clearly identified by the Separation Anxiety Test (SAT) as having insecure-avoidant or disorganized attachment representations. Children were between 7 and 11 years old (*M *= 9.25, SD* *= 1.12), with no age-difference between support conditions [*F*(1, 2)* *= 1.196, *p *= 0.312]. Twenty-four children (51%) were classified as disorganized with regard to their attachment representation and 23 (50%) as insecure-avoidant. Children with insecure-avoidant and disorganized attachment were evenly distributed among support conditions (Φ* *= 0.115, *p *= 0.732). All participants completed the TSST-C without displaying more than an expected and acceptable level of nervousness. Children in the original sample (*N *= 88) were randomly assigned to the three support conditions, with more subjects in the dog group, since originally also differences between different dogs were to be investigated. Assessment of the attachment classification resulted in ten children in the group with support by a friendly student condition, 13 children in the group with the toy dog condition, and 24 children with support by the real dog.

### Procedures

Data were collected on two different days with 1 week in between, to avoid interferences of reactions to the different assessments. On day 1, a questionnaire on the children’s pet-ownership and attachment to their own pets (see Beetz et al., 2011) and the SAT were administered.

#### The separation anxiety test

The SAT (Hansburg, 1972; Klagsbrun and Bowlby, 1976; Julius, 2009) is a projective picture task to assess attachment representation in children (age 6–12). In the German version for male children (Julius, 2009), the eight pictures show a boy who is being separated from an attachment figure for a shorter or longer period of time. The subject is asked how the child in each picture would feel, what he would think, what he would do next, and how the story would end. Transcripts of these narratives were coded for elements of secure, avoidant, ambivalent, or disorganized attachment by a reliable coder according to the system developed by Kaplan (1987). The SAT is a validated and widely used measure in attachment research with good inter-rater-reliability (93%, Wright et al., 1995; 76%, Solomon and George, 1999).

#### The trier social stress test for children

On day 2 the TSST-C (Kirschbaum et al., 1993) was conducted, aiming at inducing psychosocial stress in a standardized manner. It combines an uncontrollable situation with social evaluation by others (a social-evaluative threat; Dickerson and Kemeny, 2004). The TSST-C (“C,” adaptation of the test for children of the age 7 and older) leads to predictable significant changes in endocrinological and cardiovascular parameters and of self-assessed stress levels (e.g., Buske-Kirschbaum et al., 2003; Dorn et al., 2003; Het et al., 2009; Foley and Kirschbaum, 2010). The experimenter stops the procedure whenever a participant shows signs of strong distress.

In our study, the TSST-C was conducted in an unfamiliar classroom. In the beginning, the participant was allowed to rest for 8 min and was then given a short introduction to the procedure for approximately 2 min. After that, the child was given 5 min to get acquainted with the social supporter. Then the child was asked to stand in front of a committee of two unfamiliar adults (male and female) who explained that his task was to develop ideas of how a story, which was subsequently told by the committee, would continue. After the committee had left the room, the participant was given 5 min for preparation, before presenting his story for at least 3 min standing in front of the committee and being videotaped. Then the child was asked to perform a mathematical task for 2 min. At the end of the test the committee gave positive feedback and a short debriefing to the child and left. Then the child was led back to the other side of the room where he was allowed to relax for 30 min and to interact with the social supporter.

#### The three different support conditions

In previous research, the presence of a supportive friend or a friendly stranger was able to buffer responses of a person’s autonomous nervous system to psychological stressors (see Uchino et al., 1996; Lepore, 1998). In our study, participants were randomly assigned to one of three conditions: Support by a real dog, a toy dog the size of a small dog, or a friendly female student (control-groups). The “social supporters” were present for the entire TSST-C and the following relaxation time. The dogs were either friendly looking, trained therapy dogs, or a school-dog (Jack Russel Terrier, Norwegian Lundehund, Cavalier King Charles Spaniel, and two medium sized and longhaired mongrels). Participants were allowed to interact freely with them. The friendly student, a female between 20 and 25 years of age, with practical experience of working with children, was not allowed to help with the tasks, but only to talk to, and support the child emotionally. Only differences between the three support-groups were explored, focusing on the specific effect of support by a real dog vs. a human. No condition with “no-social support” was included at this time.

### Outcome measures

#### Assessment of stress – cortisol

Repeated measurement of salivary cortisol was employed to assess the psychophysiological reaction on the HPA-axis to the TSST-C. With a delay of a few minutes, salivary cortisol represents an equivalent of the free, non-protein-bound cortisol in plasma (Woodside et al., 1991; Gallagher et al., 2006). During the TSST-C and following relaxation time, five saliva samples (see Table 1) were collected over the course of approximately 1 h via standardized salivettes ^®^ (Sarstedt) and frozen at −20°C until analysis in the laboratory. Quantitative analysis was conducted via electro-chemical-luminescence immuno-assay (ECLIA, Cobas^®^ Roche, with the e 411 device), which can be used at concentrations between 0.5 and 1750 nmol/L, including the cortisol concentrations in human saliva of approximately 5–25 nmol/L. The manufacturer reports intra-assay variabilities of 1.5–6.1% (coefficient of variation) and inter-assay variabilities of 4.1–33.4%.

**Table 1:** **Procedures during the trier social stress test for children (TSST-C)**.

Task	Duration
Settling down, instruction	10 min
Salivette 1 = *t*1	2 min
SAM	2 min
Interaction with the social supporter	5 min
Salivette 2 = *t*2	2 min
TSST-C introduction	5 min
Preparation time	5 min
TSST-C	10 min
Salivette 3 = *t*3	2 min
Debriefing	3 min
Relaxation time 1 – possible interaction with the social supporter	10 min
Salivette 4 = *t*4	2 min
SAM	2 min
Relaxation time 2 – possible interaction with the social supporter	10 min
Salivette 5 = *t*5	2 min

*A Salivette is used for saliva sampling. SAM, Self-Assessment Manikin (description see below)*.

#### Assessment of stress – self-report

The Self-Assessment Manikin (SAM; Lang, 1980; Bradley and Lang, 1994) was used to assess the three dimensions cheerfulness, activation, and dominance (feeling of control) as emotional reactions to a situation via a non-verbal self-report. This self-report has been used with children from the age of 4 years (Caprilli and Messeri, 2006), also in combination with the TSST-C (Gunnar et al., 2009). In a pilot study we found that the children did not understand the dimension “dominance” (Beetz et al., 2011), which was therefore omitted in the current study. Five different stick figures (manikins) represent each dimension, ranging from one extreme (e.g., very sad) to the other (e.g., very cheerful). Children were asked to mark the picture that best expressed how they felt at the time, that is, before interacting with the social supporter, before the TSST-C and 15 min after its end (see Table 1).

#### Behavior

The entire test on day 2 was videotaped and coded for behaviors of the child and his interaction with the social supporter and experimenter using Noldus Observer version 5.0. Frequencies (occurrence per minute observation time) and durations of the interaction (total percentage of observation time) of a total of 49 variables were assessed, including physical contact, vocalization, locomotor parameters, and emotional expressions. Since seeking physical contact is usually a behavior indicating secure attachment and social support, and was found to be negatively correlated with cortisol levels in a previous study (Beetz et al., 2011), relevant behaviors related to physical and other social contact with the social supporter are reported.

### Data analysis

Data were analyzed with SPSS 17.00 via parametric (*T*-Test, ANOVA) and non-parametric tests (Kruskal–Wallis *H*-test, Mann–Whitney *U*-test, Friedman-test) and non-parametric correlations (*r*_s_).

To compare cortisol reactions among the three support situations, Area Under the Curve increase (AUCi), a standard in stress research, was used (Pruessner et al., 2003). AUCi indicates the increase and decrease of cortisol levels over the entire sampling time and takes into account individual differences in the initial cortisol levels of the participants (Pruessner et al., 2003). In our study, cortisol values from the salivary samples taken five times over the period of our experiment were integrated into AUCi analysis. Since cortisol data did not meet a Poisson-distribution, we resorted to non-parametric testing.

## Results

### Cortisol levels

Cortisol levels (nmol/L) differed significantly between the support conditions (*p *< 0.05) at *t*4 and *t*5 after the TSST-C and for AUCi as an indicator of stress modulation (Kruskal–Wallis *H*-Test; see Table 2). The children in the real dog condition had the lowest scores.

**Table 2:** **Mean cortisol levels (nmol/L; Salivettes at *t*1–*t*5) and AUCi (*N* = 47), Kruskal–Wallis *H*-Test (df = 2) for support condition**.

Cortisol	Real dog	Student	Toy dog	*H*-Test	
		
	Mean (SD)	Mean (SD)	Mean (SD)	χ^2^	*p*
Salivette *t*1	5.90 (4.68)	4.09 (0.89)	4.76 (2.20)	2.60	0.272
Salivette *t*2	7.61 (9.02)	6.24 (2.20)	6.79 (4.27)	0.14	0.934
Salivette *t*3	9.64 (11.07)	7.56 (4.70)	8.76 (5.61)	0.88	0.643
Salivette *t*4	5.02 (2.91)	8.02 (4.54)	7.33 (3.14)	7.03*	0.030*
Salivette *t*5	4.46 (2.67)	6.59 (3.35)	5.98 (2.73)	6.12*	0.047*
AUCi	41.65(323.3)	170.68 (155.7)	148.13 (194.5)	6.17*	0.046*

*p < 0.05.

**Figure 1:**
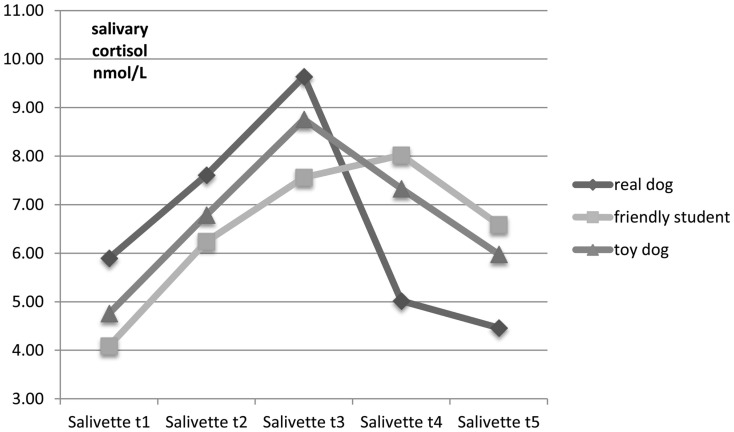
**Mean cortisol levels (nmol/L) at *t*1–*t*5 (Salivette 1–5) for each support condition**.

**Figure 2:**
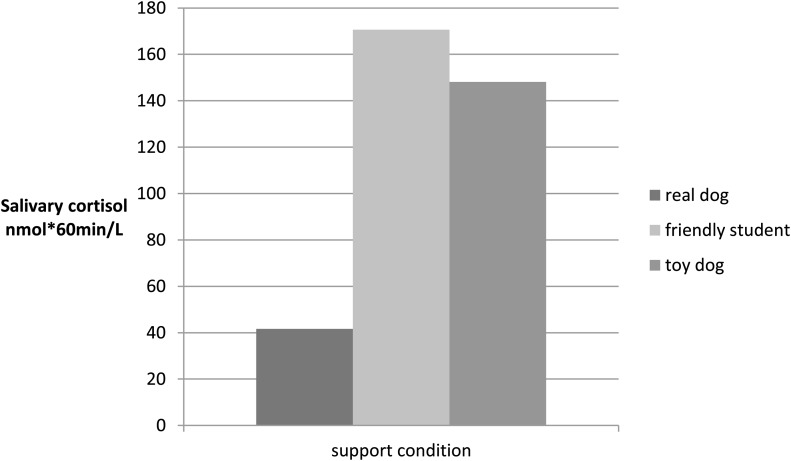
Salivary cortisol levels (nmol*60min/L): AUCi over cortisol at *t*1–*t*5 for each support condition.

Pairwise *post hoc* comparisons of the support conditions for *t*4, *t*5, and AUCi showed that the real dog group had significantly lower cortisol levels when compared to the toy dog group (Mann–Whitney U/two-tailed *p*; *t*4: *U *= 82.0, *p *= 0.019; *t*5: *U *= 93.5, *p *= 0.047; AUCi: *U *= 75.0, *p *= 0.029). A comparison of the real dog group with support by a friendly student revealed a tendency (*p *< 0.10) for higher scores in the dog group for *t*1 (*U *= 68.0; *p *= 0.088), and lower scores at *t*4 (*U *= 69.5, *p *= 0.056) and AUCi (*U *= 62.0, *p *= 0.069) and significantly lower scores (*p *< 0.05) for *t*5 (*U *= 66.5, *p *= 0.043) (see Figures 1 and 2). No significant difference was found between the toy dog and the friendly student group for cortisol levels at *t*1–*t*5 or for AUCi. Neither general pet-ownership nor owning a dog was significantly associated with cortisol modulation in the whole sample or within support conditions.

### Self-reported stress

Neither the subjects’ cheerfulness nor activation, as indicated by self-reporting via the SAM, differed between support conditions before and after the TSST-C (Kruskal–Wallis *H*-test, see Table 3). Also, a repeated measurement ANOVA with *post hoc*-tests failed to reveal any significant difference between support conditions in the self-report. A Wilcoxon-test within each support condition for the SAM-subscales showed a significant change toward less activation (more calmness) after the TSST-C in comparison to before the TSST-C only in the real dog condition (*Z *= −2.184, two-tailed *p *= 0.029). No differences in cheerfulness reached significance, nor changes in activation in the groups supported by a friendly student or a toy dog. Also, self-reported stress was independent from general pet-ownership or owning a dog.

**Table 3:** **Mean values of SAM-subscales before and after the TSST-C, Kruskal–Wallis *H*-Test (df = 2)**.

SAM	Real dog	Student	Toy dog	*H*-Test	
		
	Mean (SD)	Mean (SD)	Mean (SD)	χ2	*P*
Cheerfulness before TSST-C	4.57 (0.662)	4.40 (1.27)	4.99 (1.63)	1.47	0.929
Cheerfulness after TSST-C	4.67 (0.637)	4.70 (0.93)	4.15 (1.28)	2.49	0.289
Activation before TSST-C	2.46 (1.35)	2.40 (1.43)	2.62 (1.76)	0.04	0.980
Activation after TSST-C	1.88 (1.42)	2.50 (1.65)	2.15 (1.73)	1.42	0.493

### Behavior

Behavior was compared within subject groups over three phases: before, during and after the TSST-C.

Children spent 27% of the time before the TSST-C in body contact with the dog (holding, stroking, touching, also initiated by dog), 11% of the time in contact with the toy dog, but no time at all in physical contact with the friendly student (dog/student: *U *= 5.00, *p *= 0.001). Thus, support condition determined the amount of physical contact before, during and after the TSST-C (*p *< 0.01). During the TSST-C, children had body contact with the real dog for only 1% of the time, with the toy dog for 15% of the time and no contact at all with the friendly student. During the relaxation phase following the TSST-C, 22% of the time was spent in body contact with the dog (mostly stroking), and 21% of the time in contact with the toy dog (holding). Active touching of the social supporter by the child before the TSST-C occurred significantly more frequently with the dog than the friendly student (Mann–Whitney *U *= 5.00, *p *= 0.001; support condition: χ^2^ = 24.5, *p *= 0.001).

### Behavior and cortisol levels

In the real dog condition, we found that the more time the children spent stroking the dog before the TSST-C the greater was the drop from the highest cortisol level (either at *t*3 or *t*4) to the level at *t*5 after the end of the stressor (*r*_s_ = 0.488*, p *= 0.025). No such correlation was found for stroking the toy dog. This was not calculated for the student condition due to the low frequency of physical contact.

## Discussion

In our sample of male children with insecure or disorganized attachment, cortisol levels dropped significantly faster and to lower levels after a stressor when supported by a real dog in comparison with other support conditions (Figure 1). Similar to the results by Beetz et al. (2011), support condition did not affect peak salivary cortisol levels *during* the socially stressful situation. Self-reported, subjective stress, did not parallel salivary cortisol in the different support conditions. This is less surprising than it may seem at first, since especially persons with insecure-avoidant attachment tend to minimize or dismiss negative emotions in self-reports, also in connection with experimental stressors, while they do show expected psychophysiological stress responses (Fraley and Shaver, 1997; Mikulincer, 1998; Roisman et al., 2004; Diamond et al., 2006). Furthermore, the SAM measures each dimension only via one “item” (a row of five pictograms), which might limit its sensitivity for smaller changes in affective states. However, a within-group analysis showed a significant decrease of activation for the real dog condition only; this can be interpreted as an increase of calmness over the course of the entire procedure. Due to the preliminary character of the study and the limited sample size in the groups supported by a toy dog and a friendly student, this result might hint at possible between-group differences if sample sizes in the comparison groups were higher, in spite of the tendency to dismiss negative emotions in general in avoidantly attached individuals. However, this needs to be tested with larger samples and probably more sensitive measures.

The positive effect of the dog on post-stress relaxation fits children’s reports that in times of distress they turn to their own animals for support and generally have a trusting relationship with their companion animals (Kurdek, 2008, 2009a,b; Beetz et al., 2011). We suggest that a friendly interaction with the dog may trigger release of the hormone oxytocin, which inhibits cortisol synthesis, and thereby, also facilitates relaxation and stress regulation (de Vries et al., 2003; Beetz et al., 2011; Uvnäs-Moberg et al., 2011; Julius et al., 2013). It has indeed, been documented that friendly interactions with dogs, especially stroking them, may increase systemic oxytocin (Odendaal, 2000; Odendaal and Meintjes, 2003; Miller et al., 2009; Nagasawa et al., 2009; Handlin et al., 2011). Our assumption is supported by our finding that the stress-dampening effect of the dog was not just due to its mere presence, but actually was related to the intensity of physical contact and active stroking of the dog. Distant interaction, especially with an unfamiliar animal, is probably not as effective as physical contact in causing an increase in oxytocin levels, since pleasant touch directly triggers oxytocin release (Stock and Uvnäs-Moberg, 1998) in response to the activation of non-noxious sensory nerves stimulated by physical contact (Petersson et al., 1999; Matthiesen et al., 2001; Lund et al., 2002; Handlin et al., 2009).

Obviously most children related to the dog during the stressful procedure and were thus able to utilize it for social support, at least in the relaxation phase after the end of the stressful situation. As has been shown in previous research, interaction with animals, and even more so, with one’s own companion animal, can alleviate endocrinological and cardiovascular stress responses, very likely to a large extent mediated by oxytocin (Friedmann et al., 1983; Nagengast et al., 1997; Allen et al., 2002; Motooka et al., 2006; Cole et al., 2007; Viau et al., 2010; Beetz et al., 2011; Handlin et al., 2011; for a review see Julius et al., 2013). Since this was found in male as well as female participants, and previous research on stress responses in children found no sex-differences, it may be justified to assume similar effects in female children. However, this needs to be confirmed in further studies.

## Conclusion

We conclude that male children with insecure and disorganized attachment may profit more in regulating their physiological stress levels from the availability of a friendly dog than of a human or toy dog. Our findings are of particular relevance for understanding the underlying mechanisms of animal-assisted interventions and for explaining the additional benefit of involving an animal in pedagogic and therapeutic settings. Animal-assisted education has become increasingly popular in North America as well as in Europe, where a significant number of teachers today take their dogs with them to school. This practice to employ dogs in schools (regular and for special education and in special programs, e.g., for reading) can be justified by our results of reduced stress levels via the interaction with the dog, which supports optimal conditions for cognitive as well as socio-emotional learning. Our results hold promise for developing even more specific animal-assisted tools toward more efficient interventions for children as well as adults with insecure and disorganized attachment, who represent the majority of populations with special education needs and mental health problems (van Ijzendoorn and Bakermans-Kranenburg, 1996; Julius, 2001). However, also in regular schools nearly every second child (Ainsworth et al., 1978; Grossmann et al., 1981) has an insecure attachment representation and thus might profit more from social support by a dog than by a friendly teacher or classmate during stressful tasks like tests or giving a presentation. Furthermore, positive, stress-reducing effects of interacting with animals have been found independent of attachment representations, which suggests that probably every child (also with secure attachment) could profit from interacting with a friendly dog. In theory, securely attached children should also be able to use a friendly human for social support, but it seems obvious that establishing positive body contact, which is most effective in stress regulation, is much easier with a dog than a teacher. In pedagogic practice it could be quite beneficial for students, in particular those prone to become stressed or anxious, to be able to interact with a friendly dog, in the best case the familiar “school-dog,” before and during stressful tasks (Beetz, 2012). Potentially, the lower cortisol levels could allow for more effective executive functioning (Miyake et al., 2000) and thus even support a better performance.

## Conflict of Interest Statement

The authors declare that the research was conducted in the absence of any commercial or financial relationships that could be construed as a potential conflict of interest.
